# Chronic Pain in Older Adults: Prevalence, Risk Factors, and Consequences

**DOI:** 10.1093/geroni/igaf122.070

**Published:** 2025-12-31

**Authors:** Gillian Fennell

## Abstract

Over 100 million Americans experience chronic pain, and older adults are disproportionately affected. This symposium presents four studies using large population-based samples of older adults from three different countries to examine the prevalence, risk factors, consequences, and management of chronic pain. Using data from two generations of Framingham Heart Study participants, Felson identified a secular increase in widespread pain prevalence measured when both cohorts were in their 70s. This rising prevalence provides strong context for Limani’s examination of chronic pain’s effect on successful aging in the Canadian Longitudinal Study on Aging. Limani found that chronic pain significantly lowers respondents’ perceptions of their physical, psychological, and social wellbeing, with lower income and higher pain severity exacerbating these effects. Discerning safe and effective chronic pain management in older adults is a core research priority, particularly around opioid use. Milani investigated the interplay between sex, race/ethnicity, and cognitive impairment on self-reported opioid use from the Health and Retirement Study (HRS). After integrating Medicare Claims data, Milani will additionally explore parallel patterns of opioid prescription. Finally, Huang highlights the enduring health impact of early-life trauma using data from the Vietnam Health and Aging Study: early-life exposure to the Vietnam War increased the likelihood of later-life chronic pain. These studies leverage data from well-powered surveys to document trends in widespread pain prevalence, person-level consequences of chronic pain, disparities in opioid use, and the long-term health consequences of traumatic early-life experiences.

